# Maternal health care professionals' perspectives on the provision and use of antenatal and delivery care: a qualitative descriptive study in rural Vietnam

**DOI:** 10.1186/1471-2458-10-608

**Published:** 2010-10-14

**Authors:** Sophie Graner, Ingrid Mogren, Le Q Duong, Gunilla Krantz, Marie Klingberg-Allvin

**Affiliations:** 1 Department of Clinical Science, Obstetrics and Gynecology, Umeå University, 901 87 Umeå, Sweden; 2 Population Services/Vietnam, Hanoi, Vietnam; 3 Department of Community Medicine and Public Health, The Sahlgrenska Academy at University of Gothenburg, Gothenburg, Sweden; 4 IHCAR, Karolinska Institutet, Stockholm, Sweden; 5 School of Health and Social Sciences Dalarna University, Falun; Sweden

## Abstract

**Background:**

High quality maternal health care is an important tool to reduce maternal and neonatal mortality. Services offered should be evidence based and adapted to the local setting. This qualitative descriptive study explored the perspectives and experiences of midwives, assistant physicians and medical doctors on the content and quality of maternal health care in rural Vietnam.

**Method:**

The study was performed in a rural district in northern Vietnam. Four focus group discussions with health care professionals at primary health care level were conducted. The data was analysed using qualitative manifest and latent content analysis.

**Result:**

Two main themes emerged: "Contextual conditions for maternal health care" and "Balancing between possibilities and constraints". Contextual conditions influenced both pregnant women's use of maternal health care and health care professionals' performance. The study participants stated that women's uses of maternal health care were influenced by economical constraints and cultural norms that impeded their autonomy in relation to childbearing. Structural constraints within the health care system included inadequate financing of the primary health care, resulting in lack of human resources, professional re-training and adequate equipment.

**Conclusion:**

Contextual conditions strongly influenced the performance and interaction between pregnant women and health care professionals within antenatal care and delivery care in a rural district of Vietnam. Although Vietnam is performing comparatively well in terms of low maternal and child mortality figures, this study revealed midwives' and other health care professionals' perceived difficulties in their daily work. It seemed maternal health care was under-resourced in terms of staff, equipment and continuing education activities. The cultural setting in Vietnam constituting a strong patriarchal society and prevailing Confucian norms limits women's autonomy and reduce their possibility to make independent decisions about their own reproductive health. This issue should be further addressed by policy-makers. Strategies to reduce inequities in maternal health care for pregnant women are needed. The quality of client-provider interaction and management of pregnancy may be strengthened by education, human resources, re-training and provision of essential equipment.

## Background

The UN Millennium Goals include reducing maternal and neonatal mortality and morbidity worldwide [1]. The strategies used to achieve these goals include family planning, skilled attendance during pregnancy and childbirth, and access to emergency obstetric care [2]. Antenatal care (ANC) refers to the care of pregnant women using evidence-based interventions that are considered beneficial. WHO consider it essential that all women are offered tetanus toxoid immunisation, screening and treatment of anaemia and syphilis, and are examined for pregnancy related complications such as hypertensive disorders and mal-presentations. HIV screening is recommended in as a situational intervention, i.e. in endemic areas [3]. Client provider-interaction remains the core of clinical practice and the care offered should be acceptable for both the health care professional and the client [4,5]. Clients' health care seeking behaviour worldwide has been assumed to be the result of clients' individual characteristics, patients' identified needs, health status, patients' satisfaction, the structure of the health care system, and the external environment such as the infrastructure [6-8]. Strengthening the competence of midlevel providers and improving communicative skills can improve the quality of maternal health care [9-11].

Vietnamese society has been strongly influenced both by traditional Confucian values and (after the Communist revolution in 1945) by political actions that promote equality between males and females [12]. The development goals from the 1940's strive for equal opportunities for men and women. The strategy included among other things changes in legalization and family planning goals and promoted women's position in society [12]. This is contradicted by the Confucian tradition that states that women are inferior to men [13]. The Vietnamese system is often described as being in transition with traditional beliefs still intertwined with official strategies of equality [14]. Early marriage and childbearing have been typical characteristics of the traditional Vietnamese family [13]. According to the legislation, the minimum age for marriage is 18 years for women and 20 years for men. Political and social transition has created greater inequity between population groups, disfavouring ethnic minorities living in mountainous areas [15]. Vietnam is currently undergoing changes towards a more market-oriented provision of health care.

In Vietnam, ANC is free and mainly provided at the community health stations (CHS).The Vietnamese government recommends three ANC visits during pregnancy [7,16]. Information on factors associated with the use of ANC in Vietnam is limited, but associations between maternal education, occupation, parity, and ethnicity have been shown [7,17]. About 88% percent of women are assisted by a skilled attendant at birth [18]. Vietnam enjoys low maternal mortality ratio and neonatal mortality rate compared to other countries in the same income category or geographical area [18]. The maternal mortality ratio in Vietnam is estimated to 150 per 100 000 live births; the infant mortality is estimated to be 19 per 1000 live births [18]. Two-thirds of infants' deaths are estimated to be attributed to the neonatal period, indicating suboptimal care at birth and the first week of life. The Vietnamese government states that reducing maternal and perinatal mortality is the development target for the century. The national plan on safe motherhood for 2003 to 2010 targets adequate supply of essential drugs, educated health care professionals, identification and treatment of anaemia and prevention of mother to child transmission of HIV among other objectives [19].

Strengthening the health system is crucial to provide effective care to all women and their new born children [1]. Health care need to be clinically safe and culturally sensitive [5,20]. This may be achieved by listening to the voices of the health care providers [21].

This qualitative descriptive study explored the perspectives and experiences of midwives, assistant physicians and medical doctors on the content and quality of maternal health care in rural Vietnam.

## Method

The data collection was conducted during December 2004 in Bavi district, approximately 60 km north of Hanoi, Vietnam. Farming (rice production) and live stock breeding are the main economic activities in the district [22]. The health care system in the district includes 32 communal health stations, (CHS) three regional general clinics and one district hospital with surgical capacity. The communal health station is commonly staffed with one midwife responsible for antenatal and delivery care and one nurse. Often the head of the CHS is a medical doctor or an assistant physician responsible for the general medical care and management of the CHS. The CHS is equipped with per-oral or injection drugs, but has no capacity to perform instrumental deliveries [23]. Patients in need of surgical interventions are referred to the district hospital. Each CHS supports approximately 7500 inhabitants.

A demographic surveillance site called the FilaBavi has been functioning in the Bavi district since 1999. The FilaBavi has been described in detail elsewhere [22]. FilaBavi was used as a framework for identifying eligible participants for the study. All midwives responsible for ANC at the CHS associated with FilaBavi (n = 21) and eight medical doctors and assistant physicians were identified using the salary list (N = 29). They were invited by a written letter to participate in the study. All invited participants agreed to participate.

Four FGDs with six to eight participants in each group were conducted. The groups were homogenous with respect to profession; three FGDs were performed with midwives and one FGD with assistant physicians and medical doctors. Background information on the participants is presented in Table 1.

**Table 1:** Participants in the FGD by age, sex, profession, and years of professional experience.

FGD no	Participant no	Sex	Age (years)	Profession	Years of Professional Experience
1	1	F	37	Midwife	15
	2	F	26	Midwife	4
	3	F	23	Midwife	2
	4	F	30	Midwife	12
	5	F	24	Midwife	10
	6	F	49	Midwife	24
	7	F	36	Midwife	12

2	8	F	32	Midwife	9
	9	F	30	Midwife	7
	10	F	42	Midwife	19
	11	F	41	Midwife	17
	12	F	32	Midwife	10
	13	F	52	Midwife	30
	14	F	40	Midwife	16
	15	F	32	Midwife	9

3	16	F	47	Midwife	24
	17	F	23	Midwife	2 mths
	18	F	45	Midwife	25
	19	F	39	Midwife	7
	20	F	41	Midwife	20
	21	F	45	Midwife	20

**Mean**					**14.0 ± 8.1 years**

4	22	M	35	Medical doctor	11
	23	M	43	Assistant physician	21
	24	M	48	Assistant physician	28
	25	M	42	Assistant physician	5
	26	M	36	Assistant physician	12
	27	F	39	Medical doctor	5
	28	F	35	Assistant physician	12
	29	M	48	Assistant physician	14

**Mean**					**13.5 ± 7.8 years**

An interview guide was constructed for the first three FGD. It was revised before the FGD with medical doctors and assistant physicians, and issues related to the management of the CHS were added. The FGDs took place during the day in a conference room at one of the CHS. The FGD was moderated by one of the members of the research team (third author; Le Quyen Duong, LQD) who at the time was a female medical student. The moderator (LQD) presented open-ended questions concerning participants' perspectives and experiences related to antenatal and delivery care. First author Sophie Graner, (SG) participated in the FGD as an observer. SG had a female Vietnamese interpreter next to her who quietly translated Vietnamese into English during the FGD sessions. Field notes were taken by SG and a Vietnamese female assistant during the FGDs. All participants were informed of the aim of the study and that participation was voluntary. Each participant was guaranteed confidentiality and oral consent to use the tape recorder was obtained from all participants. The FGDs were transcribed verbatim and then translated to English. Each FGD lasted approximately 90 minutes.

Qualitative manifest and latent content analysis was applied to analyse the data [24]. The analysis aimed at finding the manifest and latent meaning of the data [25]. Content analysis is a stepwise process [26]. The data was initially read several times by SG and last author Marie Klingberg-Allvin (MKA) in order to find a sense of the whole. The data was thereafter divided into units of meaning that were condensed [25]. The condensed meaning units were abstracted and labelled with a code by SG and MKA independently. Diverging codes were re-evaluated and consensus was reached. The codes were then compared and divided with regard to similarities and differences by SG together with MKA. The codes were later grouped into subcategories and categories by the same authors, searching a more latent content for each level of abstraction. After discussing the latent meaning of the categories two main themes emerged. All the authors read, discussed and agreed on the final categorization and themes.

The study was approved by the head of the Bavi district hospital and the Medical University, Hanoi.

## Results

Two main themes emerged: "Contextual conditions for maternal health care" and "Balancing between possibilities and constraints". The themes, categories, and subcategories are presented in Table 2. Categories, subcategories, and selected quotations are presented under each theme to illustrate the findings.

**Table 2:** Themes, categories and sub-categories

Theme	Category	Subcategory
Contextual conditions for maternal health care	Facilitators and barriers to access of maternal health care	*Pregnant women's reasons for attending ANC*
		
		*Changing society*
		
		*Son preference*
	
	Organisation of maternal health care	*Routine care at ANC and at delivery*
		
		*Management of CHS and prerequisite for employment*
		
		*Suggested improvements*
	
Balancing between possibilities and constraints	Interpersonal interactions	*Midwife-pregnant woman interaction*
		
		*Husbands role during pregnancy and childbirth*
		
		*Midwife-family interaction*
		
		*Pregnant women's desire to deliver at the CHS*
	
	Health care professionals' working conditions	*Physical work environment*
		
		*HIV*
		
		*Mental work environment*

### Contextual conditions for maternal health care

Two categories are included in this theme: "Facilitators and barriers to access of maternal health care" and "Organisation of maternal health care".

#### Facilitators and barriers to access of maternal health care

Different socio-cultural factors that influence whether and when pregnant women participate in the antenatal care programme were discussed. The rate of ANC visits among women was perceived by the maternal health care professionals to be high. Economical constraints, lack of time, or a pregnant woman felt well during pregnancy were mentioned as reasons for women to sometimes neglect ANC.

*The pregnant women living in rural areas have financial and time constraints for examination [since they need to work]. I have to explain to them that they might experience complications affecting themselves and their unborn child during their pregnancy. *(Midwife)

Free iron supplementation was mentioned as a contributing factor for attending ANC. Participants had noticed a lower ANC attendance after this governmental supply was withdrawn.

*Some years ago in the ANC program we gave pregnant women iron supplements free of charge. They visited us regularly then. Now, we don't give them iron supplements, so they neglect the examinations. *(Midwife)

Women expecting their third or subsequent child were perceived to be ashamed, and it was expressed that these women tried to conceal their pregnancy.

*They expect the fourth child. When the periods don't appear on the defined day, they tell us under their breath. *(Midwife)

Improved general knowledge regarding sexual and reproductive health and the Vietnamese two-child policy were described as the main reasons for a decreased number of children per family. The pregnant women, their husbands and the extended family were perceived as keen on optimizing conditions during pregnancy.

*Because now, they only have few children. Pregnant women are taken care of from pregnancy till birth in order to have a healthy baby. Even some disadvantaged pregnant women without husband came to ask us for counselling to have a healthy baby. ... Generally, the perception and awareness [of pregnant women and their family about pregnancy] is profitable for the health of both mother and baby. *(Midwife)

The participants stated that the most important reason to continue childbearing after the stipulated two children was lack of sons. These multiparous women were considered at high risk both from a physical and psychological perspective and in need of extra care from the midwife.

*Some women who have given birth 4-5 times, but all of her babies are girls, often have psychological shocks. Some women live together with parents-in-law who are often impacted by feudal belief. These women are required to continue to give a birth until she has a son. If not, their parents-in-law will look for another wife for their husband in order to have a son to maintain the continuity of the family line. Therefore, these women are very worried. So, we need to encourage them a lot. *(Midwife*)*

#### Organisation of maternal health care

The daily responsibilities included patient mobilization, public information, examination and counselling of pregnant women, and management of labour. Women identified as high-risk patients were referred to a higher level of maternal health care either during pregnancy or at the time of birth.

*After monitoring for eight hours, the cervix is only dilated 3-4 cm. In the 9*^*th *^*hour, it's within alert time. We have to send the pregnant woman to hospital at once. This woman has prolonged labour. The partogram helps us identify the risk deliveries at an early stage and send the pregnant woman to hospital on time. *(Midwife)

*If we discover a woman with high blood pressure in this station, the best is to refer her to the hospital; that's the rule here. *(Midwife)

Lack of money and transportation were mentioned as the main obstacles for referral but also fear of medical complications or pain after surgery emerged as additional factors.

*Although my CHS is far from the hospital, we do not have access to cars. In urgent cases, we use motorbikes to take the patients to the hospital. It is frightening when an obstetrical emergency occurs during or after birth. *(Midwife)

Self-medication with drugs available in the market is common in Vietnam. Pregnant women were perceived by the participants as very knowledgeable about drugs' potential harmful effects during pregnancy. It was anticipated that pregnant women sought medical advice from health care professionals before taking medication. On the contrary, traditional herbs were generally considered as harmless.

The heads of CHS - i.e., medical doctors or assistant physicians - were responsible for the organization of the daily work and medical routines as well as the administrative tasks. The ANC and referral of pregnant women were mainly the responsibility of the midwives but supervised by the heads of CHS.

*The station head has the right to supervise the midwives in their work. If there is any problem, he has the right to discipline the midwives. *(Assistant physician)

The heads of CHS tried to organize the work so that all employees were informed of women assessed as having high-risk pregnancies. This was done through weekly meetings and labelling of medical records with coloured paperclips. The heads of CHS also stressed the importance that health care professionals needed to be available 24 hours per day.

*In my station, I try to set up some requirements for the employment of staff. If possible, we should select staff that lives near the station. I refuse to employ a candidate who lives far from the station. *(Assistant physician)

The financing of the CHS did not allow for two employees on night duty, but this problem was solved by dividing one salary into two.

*We discussed and had a lot of meetings. However, the duty allowance is for one staff; two health staff must be assigned to be on duty to assure medical safety. One salary is divided between the two health staff on duty. The staff must accept this solution. *(Assistant physician)

The need of adequate facilities, human resources and professional re-training were stressed in all FGDs. It was clear that these constraints were frustrating for the health care professionals working at CHS.

*I think we're lacking many things. The first is clinical knowledge. I think all the officers in a CHS need to be trained to carry out deliveries. The second is lack of human resources. There should be at least two officers who are trained specially in obstetrics on a night duty in each CHS. The third is [medical] instruments. There should be a standard room in each CHS. This room should be airy in the summer and cosy in the winter. The most important thing we need now is a heater, which is essential for both mothers and babies. A lot of problems appear during labour. I also need drugs, oxygen and vacuum aspirators. The officers in CHS want the government to supply us with training, human resources, and equipment. All this is essential. *(Midwife)

*To improve knowledge and professional skills, it is necessary to have short training courses every year for the staff at the communal station. All staff will then have the chance to access new guidelines and they can share the experiences and learn the new techniques to provide better primary health care to the inhabitants. *(Assistant physician)

### Balancing between possibilities and constraints

Two categories are included: "Interpersonal interactions" and "Health care professionals' working conditions".

#### Interpersonal interactions

The midwives were eager to underline their role as health care providers and the importance of pregnant women following their medical advice.

They [pregnant women] must rely on us. We tell them what we can anticipate.

*They often follow properly what we advise and never argue. *(Midwife)

The hierarchical relationship between the midwife and the pregnant woman illustrated above was contradicted by many examples of a caring attitude. Being unmarried and pregnant is socially unacceptable in Vietnam. A married woman is cared for by her husband, but a single young pregnant woman is considered vulnerable socially, usually expelled by her family and at increased risk of having a complicated pregnancy and birth.

*When a woman is pregnant, her husband takes care of her. However, the girls who are unmarried are often not cared by their families either. . . . Therefore, I take special care of them. ***(**Midwife)

In Vietnam, the legal age for marriage is 18 years for women, and pregnancy before this age was considered to be associated with obstetrical and psychosocial complications.

*Personally, I haven't met any cases like that [pregnant adolescents] in my commune. If there had been such cases, I would have been very worried for everything. Firstly, these young mothers are worried about their situation during the pregnancy. Secondly, the neighbours and relatives will have negative opinions about her pregnancy. They [the adolescents] withdraw, creating difficulties for the health professionals to communicate with them. They are very shy and don't dare to have antenatal care or vaccination. Thus, I think that they will be at a higher risk when they give birth *(Assistant physician)

It was prominent in all groups that the husbands' involvement in childbearing and birth had changed during the last years. It was described how most of the husbands were deeply concerned about their pregnant wife and supported her to take precautions to deliver a healthy and strong child. Husbands were considered important support for the women during pregnancy and childbirth, and they were often present in the delivery room to support their wives. Some husbands, however, were considered shy and the traditional view that childbearing is women's responsibility remained in the population.

*They [husbands] are actually very considerate, but regarding the childbirth, they are not able to care for their wives since they perceive the childbirth as the responsibility of women. *(Midwife)

The midwives presented different strategies to motivate women to attend sufficient number of ANC visits during pregnancy. They had a close collaboration with population counsellors in each village who reported women that were pregnant and advised them to attend ANC. Midwives described how they approached pregnant women at home in order to motivate them to have examinations.

*It is convenient because we have collaborators. They know who is pregnant because they live in the same village. . . . The collaborators go to the household if the pregnant woman has not attended ANC. We come together with them to the women's homes. *(Midwife)

A prominent opinion expressed in all groups was the pregnant women's desire to give birth at the CHS.

*They [pregnant women] mostly want to give birth in CHS because the midwives are friendly. . . . They will feel unsatisfied if we advise them to go to the hospital*. (Midwife)

In some cases, the midwives suspected pregnant women hid essential information in their obstetrical history or delayed arrival at the CHS so they could deliver at CHS. The midwives described how they found themselves pressured to carry out risk deliveries at the CHS.

*Many pregnant women try to hide their obstetric history. I was sure that one pregnant woman had had obstetrical complications earlier, but she insisted on the opposite. Then I have to tell her that I am sure her previous deliveries were difficult and she admitted to a complicated obstetrical history. *(Midwife)

Strategies were developed to avoid blame from pregnant women or their families in case of an unfavourable pregnancy outcome. Some midwives suggested that the pregnant woman had an antenatal ultrasound investigation in order detect foetal malformations before birth. In other cases the pregnant women or their relatives were sometimes asked to sign a paper that removed the health care professional of responsibility.

*I request all the women between 7*^*Th *^*and 9*^*th *^*month of pregnancy to have an ultrasound scan. I want them to find out about any foetal deformity as soon as possible. If we identify the malformation when the baby is born, the family may think that it is my mistake. *(Midwife)

#### Health care professionals' working conditions

The midwives described mental and physical constraints in their work environment. Some felt they could handle first-degree lacerations, but lacerations of higher degrees were sometimes left to heal without adequate treatment.

*I lack proper instruments for suturing. I'm only able to suture the exterior. If the interior ruptures, I can do nothing. I can diagnose interior ruptures but I have to ignore it because I don't have essential instruments for suturing. ***(**Midwife)

The health care professionals' ability to safeguard good hygiene and apply aseptic techniques during care during childbirth in order to prevent infections was limited. Shortage of appropriate gloves and sterilizers caused concern in relation to pregnant women who were HIV-positive. Fear of HIV transmission was explicit among the participants and especially problematic as they lacked the equipment to protect themselves from HIV transmission. The risk of becoming HIV infected in their daily work was acknowledged as a substantial problem:

*It's very dangerous to work in the CHS. One of my husband's friends, who is a principal of a school, said to my husband: 'Your wife works in obstetrics. We do not know if she is infected with HIV or not. She may transmit it to you.' That man made me worried. *(Midwife)

The midwives' situation was defined by lonely work, long work hours and few possibilities for collegial support even during obstetrical emergencies. Their situation was further described as lack of collegial support and mistrust of their clinical competence at debriefing sessions.

*Her family [the pregnant woman] wasted time for preparation such as transportation. She arrived at hospital late. In this case, the head of the department of obstetrics criticised me. At CHS, she [pregnant woman] has laid on the investigation table for a very short time. However, she told the head [at the hospital] that she had laid on the investigation table for 30 minutes. Unfortunately, he believed her. *(Midwife)

An altruistic attitude and a pride of being a midwife were conveyed. Their salary, however, was not considered proportionate to the responsibility, the workload, and the personal risks involved.

*Health officers at CHS like me have many difficulties at work. Sometimes I think I'm a very good health worker. *(Midwife)

## Discussion

The most prominent findings of this qualitative descriptive study were the significance of the contextual conditions for pregnant women's and health care professionals' actions and structural factors restricting the performance within antenatal and delivery care. The pregnant women's use of maternal health care was influenced by economical constraints and cultural norms that impeded their autonomy in relation to childbearing. Structural constraints within the health care system included inadequate financing of primary health care (resulting in lack of human resources), of professional re-training, and of adequate equipment.

Vietnam has undergone rapid socio-demographic changes and economical growth during the last two decades that have created inequity [17]. Our study describes how economical constraints act as a barrier for pregnant women to attend ANC. Hesitance to seek a higher level of medical care in case of an obstetrical emergency and fear of complications and pain after surgery were further described. Worldwide the aim is to reduce the patient fees for health care [1]. A national health insurance scheme in Vietnam is in place with benefits for those in paid employment and for the poor [20]. In the geographical area where the study was conducted, women were self-employed and hence not covered by the insurance scheme. A study focusing on a cash transfer scheme in India has shown the importance to target the poorest women when designing policies as well as addressing the quality of obstetrical care at the health facilities [27].

According to the participants in our study, women expecting their third child or more were exclusively women with only daughters. They were described to attend ANC late and hence considered as obstetrical risk patients for both medical and psychological reasons. This finding is coherent with another study from Vietnam where high parity and belonging to an ethnic minority was negatively associated to the use of ANC [7]. Women expecting a third child (or more) might have neglected to attend ANC in fear of being reprimanded according to the official two-child policy even though this policy was officially abolished in 2003 [7,17].

Pregnancy and childbearing are the leading cause of death among adolescents (ages 15 to 19) worldwide [28]. In the current study, adolescents were perceived as being at high obstetrical risk and midwives tried to give pregnant adolescents extra support. The midwives had the opinion that adolescents should deliver at the hospital rather than in the CHS. This was an interesting finding since there is little medical evidence for this recommendation. This finding further contrasts with the evidence-based interventions influencing the Vietnamese maternal health care in general [29]. In Vietnam, the legal age for marriage is 18 years, and plausibly the perception of young women as risk patients was more an expression of the social vulnerability these women suffer.

Vietnam is a society with a patriarchal family system based on Confucian values where women's decision making power is limited [30]. This was confirmed in the FGDs by the description of the role of the pregnant woman's husband and mother-in-law during pregnancy and childbirth. Men's influence on women's sexual and reproductive health outcomes have been increasingly recognised globally. The need to incorporate men has been highlighted when developing frameworks for sexual and reproductive health programs [31]. Male involvement has been shown to be an important strategy in improving women's reproductive health in a similar context [32]. Strengthening women's empowerment in decision making is essential to improve reproductive health [1]. The participants in our study described how men constituted important support for their pregnant wives and how the midwives encouraged men to be involved at childbirth. This and the participant's positive attitudes suggest that it is feasible to incorporate men in future reproductive health programs in Vietnam.

### Constraints to provide high quality of maternal health care

Quality of maternal health care has been neglected in many low income countries, but the perspective is changing and quality is now seen as a key element in the provision of health services in a country like Vietnam [33]. Lack of human resources remain an obstacle to an efficient health system in many countries [1]. Quality of care depends on both high technical competence, access to adequate equipment, and good client-provider interaction [8]. All participants in our study stressed lack of adequate equipment, human resources, and professional retraining as major constraints. The heads of CHS urged that the primary health level should be prioritized and funding increased. Implementation of continuous in-service training for health care professionals at all levels is essential to safeguard the clinical competence required to meet all clients' needs. Supervision and audits with feedback, rather than only written guidelines, have been identified as effective tools to improve health care professionals performance in low income settings [34].

The heads of CHC described several strategies on how to optimize the use of human resources at the health stations, but they did not provide strategies to improve specifically the client-provider interaction. Successful interpersonal relations are not only the result of the individual provider's personality, but also depend on numerous managerial decisions such as the establishment's norms, supervision, job description, and level of training [8,33]. Exchange of adequate information given by competent providers who are sensitive to the client's specific needs during pregnancy and labour are central in order to improve the quality of maternal health care [35]. In Vietnam, an individual perceived as knowledgeable (either due to academic training or age) is held in esteem. The traditional hierarchal client/provider relationship has been shown to negatively influence the quality of family planning information in another study from Vietnam [36].

Another prominent finding was the perception that pregnant women strongly desired to deliver at the CHS and they sometimes resisted being transferred to a higher medical level. As previously discussed financial situation in the family could contribute to this hesitance. An additional explanation might be lack of understanding due to inadequate information on the medical conditions upon which the decision was based. It has been estimated that only 50% of Vietnamese women are informed on the procedures during ANC and the women's involvement in decision making is uncommon [37]. In the present study, the midwives expected their pregnant women to follow the medical advice given.

#### Methodological considerations and trustworthiness

Focus group discussion is a method to gather information from specific population subgroups on a topic not well known to the investigator [38]. The method allows for the generation of rich and detailed data that usually leave the study participants perceptions intact [38]. We composed homogenous groups with regards to profession (6-8 participants) to avoid hierarchical relations [39]. After each FGD, the data was preliminary analysed and after the first three FGD with midwives, all eligible midwives in the study area had been included. A fourth FGD with assistant physicians and medical doctors was then conducted to explore any new perspectives based on professional background. However, no new information emerged in this fourth FGD and hence it was concluded that saturation of the data was obtained.

It has been suggested to divide trustworthiness into credibility, dependability, transferability and conformability [40]. We have made an attempt to provide a clear description of the data collection, data analysis, the context and the research team in order to allow the reader to assess the rigour of the study [40,41]. In the current study we argue that the degree of credibility is high due to well-organized and well-prepared focus group discussions moderated by a person with prior knowledge of the context, language, and cultural meanings related to the topic studied. The dependability was strengthened by the fact that data was collected during a relative short time (four weeks), and we do not consider that the phenomena under study changed over time. Field notes were taken by SG during the FGD. The notes were additional to the transcribed and translated data and enriched the material. The data collection was five years prior to the final analysis, which may be considered a limitation to the study and made triangulation and member checks impossible for practical reasons. Transferability was strengthened by the clear description of the data collection procedures, the setting and process of analysis. One weakness might be that the data was initially collected in Vietnamese and then translated into English and thereafter analysed. We tried to compensate for this disadvantage by confirming the results with the Vietnamese member in the research team (LQD) who also rechecked the transcription and retranslated the quotations back into Vietnamese. A multidisciplinary team with Vietnamese and Swedish researchers with substantial experience from qualitative research took part in all stages of the research process [42].

## Conclusion

Contextual conditions strongly influenced the performance and interaction between pregnant women and health care professionals within antenatal care and delivery care in a rural district of Vietnam. Although Vietnam is performing comparatively well in terms of low maternal and child mortality figures, this study revealed midwives' and other health care professionals' perceived difficulties in their daily work. It seemed maternal health care was under-resourced in terms of staff, equipment and continuing education activities. The cultural setting in Vietnam constituting a strong patriarchal society and prevailing Confucian norms limits women's autonomy and reduce their possibility to make independent decisions about their own reproductive health. This issue should be further addressed by policy-makers. Strategies to reduce inequities in maternal health care for pregnant women are needed. The quality of client-provider interaction and management of pregnancy may be strengthened by education, human resources, re-training and provision of essential equipment.

## List of abbreviations

ANC: Antenatal care; CHS: Communal health stations; FGD: Focus group discussions; GK: Gunilla Krantz; IM: Ingrid Mogren; LQD: Le Quyen Duong; MKA: Marie Klingberg-Allvin; SG: Sophie Graner

## Competing interests

No competing interests are declared. SG and MKA received planning grants from the Swedish International Development Cooperation Agency (SIDA) 2004 for conducting the study.

## Authors' contributions

SG, Ingrid Mogren (IM) and MKA created the study design. SG, IM, MKA and LQD designed the guidelines used in the data collection. SG and LQD conducted the data collection. SG and MKA coded the data. All authors agreed on the categories, subcategories, and the interpretation of the results. LQD rechecked the translation from Vietnamese to English. The manuscript was written by all authors. IM, Gunilla Krantz (GK) and MKA supervised the work. IM and MKA provided funding for the study. All authors read and approved of the final manuscript.

## Authors' information

The team consisted of SG and IM, Swedish obstetricians with research focus on maternal health care in developing countries, and MKA, a Swedish midwife with research focus on adolescents in Vietnam. GK is a Swedish medical doctor and public health researcher, and LQD is a Vietnamese medical doctor and public health researcher.

## Pre-publication history

The pre-publication history for this paper can be accessed here:

http://www.biomedcentral.com/1471-2458/10/608/prepub
